# FDG-PET/CT and Auricular Cartilage Biopsy Are Useful for Diagnosing with Relapsing Polychondritis in Patients without Auricular Symptoms

**DOI:** 10.3390/life11090956

**Published:** 2021-09-13

**Authors:** Saki Okuda, Yasuaki Hirooka, Tetsu Itami, Yuji Nozaki, Masafumi Sugiyama, Koji Kinoshita, Masanori Funauchi, Itaru Matsumura

**Affiliations:** 1 Department of Rheumatology, Kindai University Nara Hospital, Nara 630-0293, Japan; 2095C8@med.kindai.ac.jp (S.O.); m-sugi@med.kindai.ac.jp (M.S.); 2 Department of Hematology and Rheumatology, Kindai University School of Medicine, Osaka 589-8511, Japan; I-T-kindai.med@outlook.jp (T.I.); yuji0516@med.kindai.ac.jp (Y.N.); kkino@med.kindai.ac.jp (K.K.); mn-funa@med.kindai.ac.jp (M.F.); imatsumura@med.kindai.ac.jp (I.M.)

**Keywords:** relapsing polychondritis, diagnosis, auricular cartilage, biopsy, FDG-PET/CT

## Abstract

Relapsing polychondritis (RP) is a rare autoimmune inflammatory disease characterized by recurrent inflammation and destruction of cartilage. Although auricular chondritis is a characteristic finding in RP, it can be difficult to diagnose in the absence of auricular symptoms. A 64-year-old Japanese male was referred to our hospital with fever and respiratory distress. Contrast-enhanced computed tomography (CT) revealed bronchial wall thickening and we suspected RP; however, he had no auricular symptoms and did not meet the diagnostic McAdam criteria for RP, so we used ^18^F-fluorodeoxyglucose positron emission tomography/CT (FDG-PET/CT) to search for other cartilage lesions. This analysis revealed FDG accumulation not only in the bronchial walls, but also in the left auricle. Instead of a bronchial biopsy using a bronchoscope, we performed a biopsy of the left auricular cartilage, which is considered a relatively less invasive site. Even though the auricle was asymptomatic, the pathology results revealed chondritis. He was diagnosed with RP, and his symptoms rapidly improved with corticosteroid therapy. A biopsy of asymptomatic auricular cartilage may be useful in the diagnosis of RP. FDG-PET/CT is a powerful tool for the early diagnosis of RP, identifying inflammatory areas even in the absence of symptoms, and guiding the selection of appropriate biopsy sites.

## 1. Introduction

Relapsing polychondritis (RP) is a rare autoimmune inflammatory disease characterized by recurrent inflammation and destruction of the cartilage in various sites of the body, including the auricle, nose, throat, trachea, and bronchi [1]. Auricular chondritis is the most frequent presenting manifestation in patients with RP, and it is present in approximately 90% of RP patients over the course of the disease [2]. The onset of auricular chondritis is abrupt, with painful erythema and swelling of the external ear [3,4]. Destruction of the laryngeal and tracheal cartilage is a serious lesion that leads to poor prognosis [5]. RP is easy to diagnose if there is a typical RP lesion in the ear or nasal cartilage, but if the symptoms are atypical, the diagnosis may be difficult [2,6]. The progression of tracheal and bronchial chondritis due to a delayed diagnosis can lead to irreversible damage, including the collapse of airway structures. Early diagnosis and treatment are thus very important for a good prognosis.

The usefulness of 18-fluoro-2-deoxy-d-glucose (FDG) positron emission tomography/computed tomography (PET/CT) in the diagnosis of RP is established, and it has been demonstrated that asymptomatic chondritis can be identified by FDG-PET/CT [7]. We report the case of a patient with asymptomatic FDG accumulation in the auricle on FDG-PET/CT, and we describe the auricular cartilage biopsy that allowed a definitive diagnosis of RP.

## 2. Case

**Figure 1:**
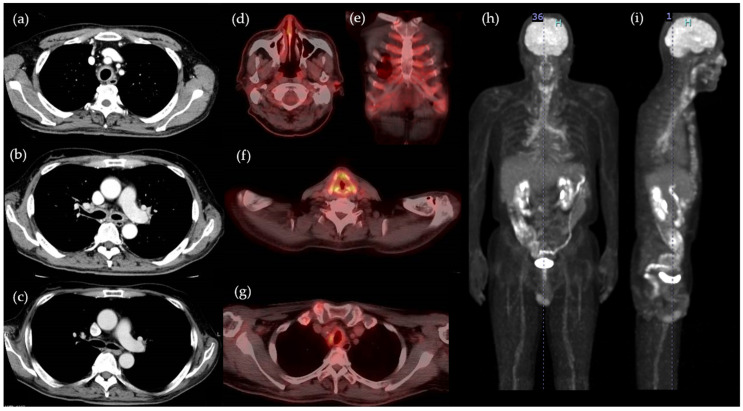
(**a**,**b**) Contrast-enhanced CT images of the patient’s tracheal and bronchial walls before the start of treatment. (**c**) Contrast-enhanced CT images findings of tracheal and bronchial walls on day 36. (**d**–**i**) FDG-PET/CT findings of the left auricle, costal cartilage, larynx, and tracheal and bronchial walls before the start of treatment.

A 64-year-old Japanese man presented to a nearby hospital with a 4-month history of fever, malaise, chest pain, and dyspnea on exertion. Blood tests indicated elevated C-reactive protein (CRP), and contrast-enhanced CT scan indicated thickening of the tracheal and bronchial walls with contrast enhancement (Figure 1a,b). He was referred to our hospital for further examination and was admitted. He had undergone an oral treatment for pulmonary tuberculosis at the age of 33, he was diagnosed with diabetes at the age of 61, and was taking metformin and saxagliptin. There was no family history of connective tissue diseases. He had no history of smoking or drinking. He did not have a fever, but he had a cough with sputum and was classified as having MRC grade 3 dyspnea. The physical examination revealed no redness or swelling of the auricles, no tinnitus, no hearing loss, and no joint pain. The results of the laboratory investigation on admission are shown in Table 1. The blood tests showed anemia, high CRP, and low albumin levels. Autoantibodies were positive for anti-centromere antibody and proteinase3-anti neutrophil cytoplasmic antibody (PR3-ANCA), but there were no symptoms such as Raynaud’s phenomenon or skin sclerosis to suggest scleroderma, and there was no skin rash, peripheral neuropathy, or pulmonary findings to suggest ANCA-associated vasculitis. Tumor markers such as CEA and CA19-9 were negative. A urinalysis showed urine protein 2+. The results of sputum and blood cultures were negative. Respiratory function tests showed a decreased forced expiratory volume in one second of 59.2%.

**Figure 2:**
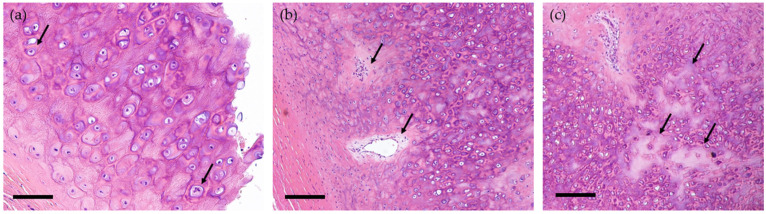
Pathological findings for the auricular cartilage. Hematoxylin and eosin (H&E) staining. (**a**) Neutrophils (*arrows*). Original magnification: ×200. Scale bar, 20 μm. (**b**) Cartilaginous infiltration of inflammatory cells (*arrows*). Original magnification: ×100. Scale bar, 40 μm. (**c**) Calcification (*arrows*). Original magnification: ×100. Scale bar, 40 μm.

We suspected relapsing polychondritis, but the patient’s case did not meet the McAdam criteria for RP. To further investigate the lesions in RP, we performed FDG- PET/CT, which revealed FDG accumulation not only in the tracheal and bronchial walls, but also in the left auricle, larynx, and costal cartilage (Figure 1d–g). A biopsy of the left auricular cartilage was performed to confirm the diagnosis. The auricle was asymptomatic, but the pathological results showed cartilaginous infiltration of inflammatory cells and calcification in line with the presence of osteochondritis (Figure 2). Based on these results, we made the diagnosis of RP. We initiated treatment with prednisolone (65 mg daily), which induced rapid improvements of the symptoms including respiratory distress on day 3 after the start of the regimen. Urinary protein became negative on day 5. A blood test on day 15 showed that the patient’s CRP level had decreased to the reference level, and a contrast-enhanced CT scan on day 36 demonstrated that the wall thickening of the trachea and bronchi had improved (Figure 1c). The serum collected at the time of the patient’s admission was observed to be positive for anti-type II collagen antibodies. Concomitant oral methotrexate (MTX) (8 mg once a week) was begun on day 47, and the patient was discharged on day 55 after the above-described treatment began.

## 3. Discussion

RP is an autoimmune disease that affects all types of cartilage, including elastic cartilage in the ears and nose, hyaline cartilage in peripheral joints, fibrocartilage at axial sites, and cartilage in the trachea and bronchi [2]. RP can also cause inflammation of other proteoglycan-rich structures such as the eyes, heart, blood vessels, and inner ear [2]. Auricular chondritis, the most common feature of RP, is the initial manifestation in 20% of cases [3], and it typically presents as abrupt inflammation of the bilateral auricles with pain, redness, and swelling. Laryngotracheobronchial involvement is seen in 30–50% of patients with RP, and RP patients with significant laryngotracheobronchial involvement have a poor prognosis [5].

It has been reported that the conditions of approximately 30% of RP patients are associated with other autoimmune diseases [8,9]. In the present case, anti-centromere antibodies and PR3-ANCA were positive, but there were no symptoms such as Raynaud’s phenomenon or skin sclerosis suggesting scleroderma, and no findings such as skin rash, peripheral neuropathy, or pulmonary findings suggesting AAV.

The diagnosis of RP is based on typical clinical findings. According to McAdam et al., RP can be diagnosed if three or more of the following six clinical features are present: bilateral auricular chondritis, nonerosive inflammatory polyarthritis, nasal chondritis, ocular inflammation, respiratory tract chondritis, and audiovestibular damage [1]. Damiani and Levine expanded the spectrum of the diagnostic criteria to include the presence of at least one McAdam criterion and positive histological findings, or the presence of two McAdam criteria and a positive response to the administration of a corticosteroid or dapsone [10]. Michet et al. also proposed a modified version of the McAdam criteria for diagnosis. RP is diagnosed if inflammation is identified in two of the three cartilages of the auricle, nasal, or laryngotracheal sites, or if inflammation is identified in one of the above cartilages plus two of the following findings: ocular inflammation, hearing loss, vestibular dysfunction, and seronegative arthritis [11]. A cartilage biopsy is not essential for diagnosis, but its findings may contribute to the diagnosis in cases of atypical clinical presentation [2,10,11].

In most cases, corticosteroids are the mainstay of RP treatment [2,8]. Immunosup-pressive agents such as azathioprine, MTX, cyclophosphamide, and cyclosporine may be administered to patients who are unresponsive to corticosteroids or who require a corticosteroid-sparing effect [2,8].

Bilateral auricular chondritis is characteristic of RP, but in patients with unilateral or absent symptoms, the diagnosis of RP can be difficult. In a case series of 66 patients, the time between the medical attention for related symptoms and diagnosis was 2.9 years [2]. Patients with ear- or nose-related symptoms as the first manifestation of RP have a shorter time to diagnosis, whereas patients with respiratory symptoms as the first manifestation of RP have a longer time to diagnosis [6]. Progression of the disease due to delayed diagnosis can lead to irreversible damage, including the collapse of airway structures. Delayed diagnosis can worsen the prognosis, and early diagnosis is very important.

In our patient’s case, early diagnosis was difficult because there were no typical auricular symptoms and cartilage abnormalities were identified only in the trachea and bronchi. Of the six clinical features listed in McAdam’s diagnostic criteria, our patient had only respiratory tract chondritis, and therefore did not meet this diagnostic threshold. According to the diagnostic criteria of Damiani and Levine, our patient required confirmation of chondritis by histopathological findings for the diagnosis of RP. The contrast-enhanced CT and FDG-PET/CT results in the present patient’s case suggested inflammation of the trachea and bronchial wall, and a biopsy at those sites by bronchoscopy was considered as an option to reach the diagnosis of RP [12]. However, the positive rate of tracheobronchial biopsies by bronchoscopy is low, and the procedure may exacerbate mucosal edema and cartilage inflammation [13,14]. It has been noted that bronchoscopy can cause an acute flare of RP, leading to airway edema, bronchospasm, bleeding, respiratory failure, and death [15].

The usefulness of FDG-PET/CT in the diagnosis of RP has been established [7,13,14,16,17,18,19]. Lei et al. reported that the presence of two or more symmetrically distributed cartilages or joints with high-FDG-uptake lesions strongly indicates RP [18]. It was also noted that targeted biopsies of the lesions with high FDG uptake had a high positive rate [18]. RP patients with auricular symptoms have been shown to have a high rate of positivity in auricular cartilage biopsy (approximately 90%) regardless of whether there is any accumulation on FDG-PET/CT [14]. In contrast, even when there was a high FDG accumulation on FDG-PET/CT, the positive rate of tracheal and bronchial biopsies on bronchoscopy was as low as 10.5% [14]. In patients with inflammation of the tracheobronchial tree but no symptoms in the ears or nose, as in the present case, a tracheobronchial cartilage biopsy can be considered, but the low positivity rate of such a biopsy and the risk of complications are drawbacks.

FDG-PET/CT may reveal FDG accumulation in cartilage at asymptomatic sites in RP patients [7]. In the absence of symptoms in a relatively safe and easily biopsied site such as the auricle, we suggest performing FDG-PET/CT. In our patient’s case, the presence of cartilage lesions in multiple sites on FDG-PET/CT strongly suggested the possibility of RP. In addition, FDG accumulation was observed in the auricle despite the absence of symptoms, and we were able to select the auricle as the biopsy site, which is relatively less invasive and has a higher positive rate. Since positive histological findings were obtained from an asymptomatic auricle, this case met Damiani and Levine’s diagnostic criteria and led to early therapeutic intervention. Importantly, in patients who require histologic findings for the diagnosis of RP, it is worthwhile to select the auricular cartilage as the biopsy site even in the absence of auricular symptoms.

To date, there have been few reports of biopsies of asymptomatic auricular cartilage leading to the diagnosis of RP. Because RP is characterized by recurrent episodes of inflammation of cartilage tissue, we believe that there are cases of subclinical auricular chondritis at the time of biopsy or in the past. As in the present case, asymptomatic FDG accumulation in the auricle on FDG PET/CT would guide the choice of biopsy of the auricular cartilage.

Early diagnosis is very important because early therapeutic intervention for RP may delay or prevent irreversible cartilage destruction. Although the high cost is an issue, FDG-PET/CT is very useful for early diagnosis because it can reveal multiple organ involvement and help to determine the preferred biopsy site. In cases in which the patient’s symptoms and other modalities do not lead to the diagnosis of RP, or when the lesion is located in only one region (e.g., trachea/bronchus) and a biopsy is considered invasive, FDG-PET is indicated to achieve an early diagnosis. Even when FDG-PET/CT cannot be performed, we believe that a biopsy from an asymptomatic auricle is an important examination that may provide a definitive diagnosis. Although McAdams’ diagnostic criteria are helpful, cases of clinical RP, like this case, still require an aggressive diagnostic approach and treatment to avoid irreversible end organ damage.

## 4. Conclusions

A biopsy of asymptomatic auricular cartilage may be useful in the diagnosis of RP. FDG-PET/CT may identify the inflamed area even in the absence of symptoms and can guide the biopsy to less invasive sites such as auricular cartilage.

## Figures and Tables

**Table 1:** The patient’s laboratory test results.

Complete Blood Counts:	Value	Reference Interval	Biochemistry	Value	Reference Interval
White blood cells	8280/μL	3300–8600	Aspartate transaminase	6 U/L	13–30
Neutrophils	77.7%	38–77	Alanine aminotransferase	7 U/L	10–42
Lymphocytes	11.0%	20.2–53.2	Lactate dehydrogenase	96 U/L	124–222
Monocytes	9.1%	2.7–9.3	Creatinine kinase	20 U/L	59–248
Eosinophils	2.5%	0.2–4.1	HbA1c	8.3%	4.9–6.2
Basophils	0.3%	0.2–1.3	TSH	1.15 μIU/mL	0.5–5
Red blood cells	314 × 10^4^/μL	435 × 10^4^–555 × 10^4^	Free thyroxine	1.4 ng/dL	0.9–1.7
Hemoglobin	8.4 g/dL	13.7–16.8	**Immunology:**		
Hematocrit	27.20%	40.7–50.1	Rheumatoid factor	Negative	0–15
Platelets	37.3 × 10^4^/μL	15.8–34.8	Antinuclear antibody	Negative	Negative
**Urinalysis:**			Anti-ds-DNA antibody	Negative	Negative
Protein	2+	–	Anti-centromere antibodies	20.4 Index	<10
Creatinine	1.14 g/day		PR3-ANCA	3.2 U/mL	0–3.4
Estimated urine protein	0.22 g/day	<0.15	MPO-ANCA	Negative	0–3.4
**Biochemistry:**			CH50	71.6 U/mL	11–31
CRP	11.2 mg/dL	0–0.14	IgG	1627 mg/dL	861–1747
Blood urea nitrogen	22 mg/dL	8–20	IgA	408 mg/dL	93–393
Creatinine	0.58 mg/dL	0.65–1.07	IgM	152 mg/dL	33–183
Total protein	7.0 g/dL	6.6–8.1	IgE	149 IU/mL	0–232
Albumin	2.4 g/dL	4.1–5.1	**Tumor markers:**		
Total bilirubin	0.2 mg/dL	0.4–1.5	CEA	2.6 ng/mL	0–5
γ-glutamyltransferase	14 U/L	13–64	CA19-9	4 ng/mL	0–37

## Data Availability

The data presented in this study are available on request from the corresponding author.
